# Construction and Comparative Analyses of Highly Dense Linkage Maps of Two Sweet Cherry Intra-Specific Progenies of Commercial Cultivars

**DOI:** 10.1371/journal.pone.0054743

**Published:** 2013-01-31

**Authors:** Carolina Klagges, José Antonio Campoy, José Quero-García, Alejandra Guzmán, Levi Mansur, Eduardo Gratacós, Herman Silva, Umesh R. Rosyara, Amy Iezzoni, Lee A. Meisel, Elisabeth Dirlewanger

**Affiliations:** 1 Universidad Andrés Bello, Centro de Biotecnología Vegetal, Facultad de Ciencias Biológicas, Santiago, Chile; 2 INRA, UR419, Unité de Recherches sur les Espèces Fruitières (UREF), Centre de Bordeaux, Villenave d’Ornon, France; 3 Estación Experimental La Palma, Facultad de Agronomía, Pontificia Universidad Católica de Valparaíso, Quillota, Chile; 4 Laboratorio de Genómica Funcional y Bioinformática, Departamento de Producción Agrícola, Facultad de Ciencias Agronómicas, Universidad de Chile, Santiago, Chile; 5 Department of Horticulture, Michigan State University, East Lansing, Michigan, United States of America; 6 INTA-Universidad de Chile, Santiago, Chile; CIRAD, France

## Abstract

Despite the agronomical importance and high synteny with other *Prunus* species, breeding improvements for cherry have been slow compared to other temperate fruits, such as apple or peach. However, the recent release of the peach genome v1.0 by the International Peach Genome Initiative and the sequencing of cherry accessions to identify Single Nucleotide Polymorphisms (SNPs) provide an excellent basis for the advancement of cherry genetic and genomic studies. The availability of dense genetic linkage maps in phenotyped segregating progenies would be a valuable tool for breeders and geneticists. Using two sweet cherry (*Prunus avium* L.) intra-specific progenies derived from crosses between ‘Black Tartarian’ × ‘Kordia’ (BT×K) and ‘Regina’ × ‘Lapins’(R×L), high-density genetic maps of the four parental lines and the two segregating populations were constructed. For BT×K and R×L, 89 and 121 F_1_ plants were used for linkage mapping, respectively. A total of 5,696 SNP markers were tested in each progeny. As a result of these analyses, 723 and 687 markers were mapped into eight linkage groups (LGs) in BT×K and R×L, respectively. The resulting maps spanned 752.9 and 639.9 cM with an average distance of 1.1 and 0.9 cM between adjacent markers in BT×K and R×L, respectively. The maps displayed high synteny and co-linearity between each other, with the *Prunus* bin map, and with the peach genome v1.0 for all eight LGs (LG1–LG8). These maps provide a useful tool for investigating traits of interest in sweet cherry and represent a qualitative advance in the understanding of the cherry genome and its synteny with other members of the *Rosaceae* family.

## Introduction

All cherry species belong to the *Cerasus* subgenus of the *Prunus* genus, within the *Rosaceae* family. Sweet cherry (*Prunus avium* L.) is an economically important crop that includes cherry trees cultivated for human consumption and wild cherry trees used for their wood, also called mazzards [1]; [2]. The majority of cultivated cherry trees belongs to the diploid (2n = 2x = 16) sweet cherry and allotetraploid (2n = 4x = 32) sour cherry (*P. cerasus* L.) species. Sweet cherry and the tetraploid ground cherry (P. *fruticosa* Pall., 2n = 4x = 32) are believed to be the parental species that gave rise to sour cherry [1]; [2]; [3]; [4].

Traditionally, the main sweet cherry breeding objectives have been to select for large, attractive and good-flavoured fruits, a short juvenile phase, abundant and consistent yields, reduced susceptibility to fruit cracking, self-compatibility and improved resistance or tolerance to diseases such as bacterial canker induced by *Pseudomonas mors* pv. *prunorum* and *P. syringae* [3]; [5]; [6]; [7]; [8].

Due to rapid climate change and the noteworthy reduction of chilling accumulation observed and/or predicted for several temperate zones [9], concern about climatic adaptation in temperate fruit trees has arisen [10]. Sweet cherries are cultivated commercially around the world in temperate, Mediterranean and even subtropical regions. In order to break bud dormancy, sweet cherries need to meet minimum chilling requirements in the autumn and winter [11]. Most fruit tree species are long-lived perennials, lasting more than 20 years, which implies that successful cultivars must be able to perform well despite changing climatic conditions. For this reason, climatic adaptation has become one of the major breeding objectives for many fruit crops and climatic adaptation of the parental plant material precedes breeding for commercial fruit qualities [12]. However, the long juvenile period (four to five years) in sweet cherry represents a drawback for quick and efficient breeding. Reducing the time needed to breed temperate fruit trees will be important to develop new cultivars that are able to face the challenges associated with temperate fruit production in a changing global environment. Marker-assisted breeding approaches based on genetic maps have the potential to save time and resources needed to select new sweet cherry cultivars.

The recent release of the peach genome v1.0 by the International Peach Genome Initiative [13]; [14], (GDR database: http://www.rosaceae.org/peach/genome) and the high synteny among *Prunus* species [15] will facilitate the characterization of genes responsible for agronomically important traits within this family. In cherry, maps from an inter-specific cross, *P. avium* ‘Napoleon’ × *P. nipponica,* have been developed [16]. From intra-specific crosses, Dirlewanger et al. [17] have developed a map from elite cultivars ‘Regina’ × ‘Lapins’, whereas Olmstead et al. [18] have constructed a map from the great-grandparent of ‘Lapins’ (cultivar ‘Emperor Francis’), and the wild forest cherry ‘New York 54′. More recently, Cabrera et al. [19] presented a consensus sweet cherry map constructed from four progenies using Rosaceae Conserved Orthologous Set (RosCOS) SNP and SSR markers. Despite the potential usefulness of genetic linkage maps for cherry, highly dense linkage maps for commercial sweet cherry cultivars have not yet been constructed. With the exception of the SNP-based map that we have recently reported [19], the majority of the cherry linkage maps have been based on low throughput molecular markers such as SSRs, AFLPs and isoenzymes. High throughput SNP genotyping technology has become available for some plant species. BeadChip or Infinium II technologies have been developed by consortiums or commercially for apple [20], peach [21], cherry, maize, tomato and *Vitis* [22], minimizing dramatically genotyping costs and facilitating the production of high density SNP maps. The RosBREED cherry 6K SNP array v1 for use with the Illumina Infinium® system developed by the RosBREED consortium [23] for both diploid sweet cherry and allotetraploid sour cherry provides a new avenue for genome scanning capability in sweet and sour cherry. This publicly available genomics resource represents a significant advance for marker-locus-trait discovery and further research about the genome structure and diversity in this diploid-tetraploid crop set [23].

In this study, we develop highly dense linkage maps of two intra-specific progenies generated from the crosses of commercial cultivars: ‘Black Tartarian’×’Kordia’ (designated as BT×K) and ‘Regina × Lapins’ (designated as R×L), carried out in Chile and France, respectively, using the new RosBREED cherry 6K SNP array v1. To our knowledge, these are the first genetic maps using this new SNP genotyping resource developed in sweet cherry. The maps were compared with the *Prunus* bin map [24] and the peach genome v1.0 to assess synteny and co linearity between the studied progenies and the bin map and the peach genome v1.0. These maps provide a new framework for sweet cherry genomic information for the international community and new opportunities to discover QTLs associated with agronomical traits in this and related species, based upon the haplotype segregation in the progeny.

## Materials and Methods

### Plant Material

Two F_1_ mapping populations were developed in Chile and France from parents with contrasting phenotypes of breeding significance (Table 1) [25]; [26]; [27]. A total of 210 progeny seedlings from intra-specific crosses were used in this study. Of the 210 individuals, 89 seedlings were from ‘Black Tartarian’ × ‘Kordia’ (BT×K) grown at Quillota, located 120 km North from Santiago, Chile, whereas 121 were from ‘Regina’ × ‘Lapins’ (R×L) and raised at Toulenne, located 30 km South-West from Bordeaux, France.

**Table 1:** Origin and characteristics of the four parental cultivars used to perform the two mapping progenies.

Cultivar	Parental cultivars	Country of origin	Main characters	Reference
Black Tartarian	U × U	United Kingdom	Medium chill, early flowering, self-incompatible (S1S2), low firmness, low sugar content, small fruit, acid fruit.	[25]; [26]
				
Kordia	U × U	Czechoslovakia	High chill, late flowering, self-incompatible (S3S6), high firmness, cracking resistance, high sugar content, large fruit, pitting resistance.	[25]; [26]
Regina	Schneiders × Rube	Germany	High chill, late flowering and ripening, self-incompatible (S1S3), cracking tolerance, sweet, large fruit, slightly acid.	[27]
Lapins	Van × Stella	Canada	Medium chill, early flowering, self-compatible (S1S4’), high productivity, low susceptibility to cracking, sweet, large fruit, very slightly acid.	[27]

U: Unknown.

The field studies performed did not involve endangered or protected species, but cultivated sweet cherry trees. In addition, all the trees used in this study belong to public institutes and do not need any specific permit to be used for the analyses described in this paper.

### DNA Extraction

Leaf material was frozen in liquid nitrogen and stored at −80°C for later use. Genomic DNA was extracted from the frozen tissue using the DNeasy® plant kit (Qiagen) according to the manufacturer’s instructions. Genomic DNA was quantified using Accublue™ dsDNA quantification kit (BIOTIUM) according to the manufacturer’s instructions for the BT×K progeny, whereas for the R×L progeny, genomic DNA was quantified using spectrophotometry Nanoview (GE Healthcare) and fluorimetry Quant-iT™ Picogreen® (Invitrogen) according to the manufacturer’s instructions. Fifteen µl of DNA with a concentration between 50 ng/µl –75 ng/µl was used for subsequent analyses.

### Genetic Marker Analysis

#### SNPs

Both sweet cherry mapping populations and their respective parents were genotyped using the RosBREED cherry 6K SNP array v1 that contains 5,696 SNPs of which 76% and 24% of the SNPs were chosen to target the sweet and sour cherry genomes, respectively [23]. SNPs were obtained from re-sequencing of a detection panel containing 16 sweet and eight sour cherry accessions. The accessions were founders, intermediate ancestors or important parents used in U.S. breeding programs [23]. SNPs were detected using *SoapSNP* [28] (SoapSNP: http://soap.genomics.org.cn/soapsnp.html) and validated by GoldenGate® assay [29]. Genotype differences were recorded in the iSCAN platform and SNP genotypes were determined using Genome Studio Genotyping Module (Version 1.8.4, Illumina, [30]; [31]; [32]) as described in Peace *et al*. [23]. All DNA samples were above the GenCall threshold of 0.15 and were therefore all used in further analyses [33]. Initial clustering was done using Gentrain2, a GenomeStudio build-in clustering algorithm ([30]; [31]; [32]). Following the clustering by Gentrain2, all SNPs were visually examined for appropriateness of clustering, cluster separation, number of clusters, presence of null alleles and paralogs along with a check on whether the progenies follow expected genetic ratios and presence of genotyping errors. A SNP was considered “failed” if that have overlapping clusters or ambiguous clusters which cannot be improved by even manual clustering, more than 3 clusters suggesting presence of paralogs and very low call frequency, and positive outlier in terms of Mendelian inheritance error rates (e.g. parent-child or parent-parent-child) [33]. The failed SNPs were not used in the mapping process.

The RosBREED cherry 6K SNP array v1 markers used in this work were deposited in NCBI’s dbSNP repository available at www.ncbi.nlm.nih.gov/projects/SNP [34] and each SNP was given a unique accession number that starts with the prefix ss (SNPs NCBI ss# database names). More information associated with these SNPs is available at the Genome Database for Rosaceae (GDR; www.rosaceae.org [35]). The location of the markers on the physical map of the peach genome v1.0 as well as their positions on the genetic maps of BT, K, BT×K, L, R and R×L are available in Table S1.

The MAF (minor allele frequency) distribution of SNPs heterozygous in each of the four mapping parents (‘Black Tartarian’, ‘Kordia’, ‘Regina’ and ‘Lapins’) was determined and then compared with a germplasm collection of 269 sweet cherry selections used by Peace *et al.* [23]. Similarly, the distribution and physical spacing (Mbp) of SNP heterozygocity along the eight cherry chromosomes for the four linkage mapping parents was compared with polymorphic SNP markers identified in the germplasm collection of the 269 sweet cherry selections [23].

#### Linkage mapping

Linkage analysis was performed using JoinMap® 4.0 for a cross-pollinated progeny [36]. Markers were coded according to the heterozygous in one (*<nn × np>*, *<lm × ll>*) or both parents (<*hk × hk>*) [36]. Map constructions were performed following a ‘Two-Step’ strategy [37].

Firstly, parental maps were constructed using only heterozygous markers for one parent (class *<nn × np>* and *<lm × ll>*, less ambiguous in tracing origin of allele than <*hk × hk>* class). ‘Suspect Linkage’ (recombination frequency >0.6) and ‘Genotype Probabilities’ (-Log10 (P) >2.0) tools were used to identify mis-grouped markers and double recombination events, respectively. Spurious double crossovers were considered as missing value while revising the map. Chromosome painting was performed for each linkage group of the four parents to verify the double recombination events. Double recombination events in narrow genetic distances were omitted from subsequent mapping analyses. The *χ^2^* (chi-square) goodness-of-fit test was used to evaluate the discrepancy with the expected segregation ratios. In a first step, all segregating markers were used for initial mapping. In a second step, isolated markers showing segregation distortions different from expected Mendelian ratios, with a probability lower than *p* = 0.01 were excluded from further analyses. The grouping and ordering of markers was done using JoinMap® 4.0’s build algorithm for cross pollinated species, a likelihood based weighted least square procedure (with the squares of the LODs as weights) as described by Stam [38]. The inspection of proper assignment of a marker to a group was done by calculating the Strongest Cross Link (SCL) parameter. The mapping procedure implemented in JoinMap consisted of iterative process: (a) adding loci one by one starting from most informative and each added marker locus’s best position is searched by comparing the goodness-of-fit of the calculated map for each tested position (b) when the goodness of fit measure decreases sharply (i.e. difference known as jump) or gives negative estimates in the map the locus is removed and process is continued till first round is completed [38]. Second and third round accommodate the marker loci that are previously removed without the constraints of maximum allowed jump level or negative values. Maps were calculated iterating different LOD values in order to minimize the Mean Chi-square contributions. LOD thresholds of six and three were used for grouping and mapping, respectively. Kosambi’s mapping function [39] was used to convert recombination frequency into map distance. Although peach physical map based on peach physical genome v1.0 provided a reference for comparisons of the linkage map output, no fixed order was forced based on peach physical positions. MapChart [40] was used to draw the linkage maps and make group-wise comparisons between maps. Secondly, an integrated map of each progeny was undertaken including heterozygous markers in both parents (<*hk × hk*>) to decrease large gaps in parental LGs. New parental maps were developed including (<*hk × hk*>) markers as previously described and integrated using the “Combine Groups for Map Integration” function of JoinMap® 4.0 to produce the combined maps.

## Results

### Marker Polymorphism

When each parent cultivar was considered separately, the percentages of heterozygous, homozygous and failed SNP markers were similar among the four parents (Table 2). The proportion of markers heterozygous within each parent was 11.1% in ‘Black Tartarian’, 9.2% in ‘Kordia’, 10.6% in ‘Regina’ and 9.0% in ‘Lapins’ (Table 2). The percentage of homozygous SNPs ranged from 83.9% for ‘Black Tartarian’ to 86.4% for ‘Lapins’ (Table 2). The SNP failure rate was also similar among parents 5% in ‘Black Tartarian’ and ‘Kordia’, 4.8% in ‘Regina’ and 4.5% in ‘Lapins’ (Table 2).

**Table 2:** Number of heterozygous, homozygous and failed SNPs for each mapping parent.

	Heterozygous		Homozygous		Failed	
	Total	%	Total	%	Total	%
Black Tartarian	634	11.1	4780	83.9	282	5.0
Kordia	526	9.2	4888	85.8	282	5.0
Regina	603	10.6	4819	84.6	274	4.8
Lapins	515	9.0	4926	86.5	255	4.5

**Figure 1:**
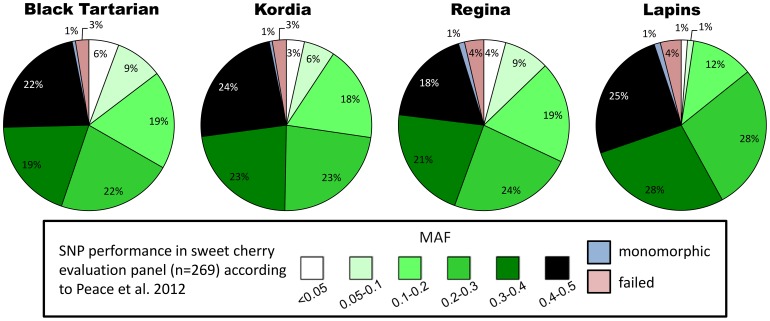
Minor allele frequency (MAF) distribution of heterozygous SNP for each four mapping parent cultivars. Percentages of heterozygous SNPs for each of the four mapping parent cultivars (‘Black Tartarian’, ‘Kordia’, ‘Regina’ and ‘Lapins’) that fell within six previously determined minor allele frequency (MAF) classes based on an analysis of 1825 polymorphic SNPs genotyped using 269 sweet cherry accessions [23]. The percentages of the 1825 SNPs that were monomorphic or failed for each parent are also indicated.

The MAF distribution of SNPs heterozygous in each of the four mapping parents was similar to the distribution observed within a germplasm collection of 269 sweet cherry selections used by Peace *et al* [23] (Figure 1). A similar proportion of SNPs for each of the MAF categories were observed. The majority of the heterozygous SNP markers (63% in ‘Black Tartarian’ and ‘Regina’, 70% in ‘Kordia’, 81% in ‘Lapins’) have been identified previously as having a MAF>0.2 based on MAFs previously determined in the collection used by Peace *et al*. [23] (Figure 1). A few of the heterozygous SNPs (2–15%) have been identified previously as having a MAF<0.1 (Figure 1). Interestingly, 4 to 5% of SNPs that were monomorphic or failed in the evaluation panel were polymorphic in the parents. Most of the heterozygous SNPs in the parent cultivars correspond to SNPs that were identified from the sweet cherry genomic sequences (87.9% in ‘Black Tartarian’, 91.1% in ‘Kordia’, 88.7% in ‘Regina’ and 88.9% in ‘Lapins’). Additionally, a significant number of the heterozygous SNPs in the parental cultivars (11.5% in ‘Black Tartarian’, 8.7% in ‘Kordia’, 10.1% in ‘Regina’ and 10.5% in ‘Lapins’) were in the SNPs that have been classified previously as part of the sour cherry *avium* subgenome. A lower number of SNPs correspond to SNPs that have been classified previously as part of the sour cherry *fruticosa* subgenome (0.6% in ‘Black Tartarian’, 0.2% in ‘Kordia’, 1.2% in ‘Regina’ and 0.6% in ‘Lapins’).

Distribution, physical spacing (Mbp) and genetic distance (cM) of SNP heterozygosity along the eight cherry chromosomes for the four linkage mapping parents (‘Black Tartarian’, ‘Kordia’, ‘Regina’, and ‘Lapins’) compared to polymorphic SNP markers identified in the 269 sweet cherry selections [23] are shown in Figures S1 and S2.

Then, by analyzing the two mapping populations, of the 5,696 SNP markers from the RosBREED cherry 6K SNP array v1, 935 (16.4%) and 831 (14.6%) were segregating in BT×K and R×L, respectively.

### High-density Linkage Maps

#### Parental linkage maps

**Figure 2:**
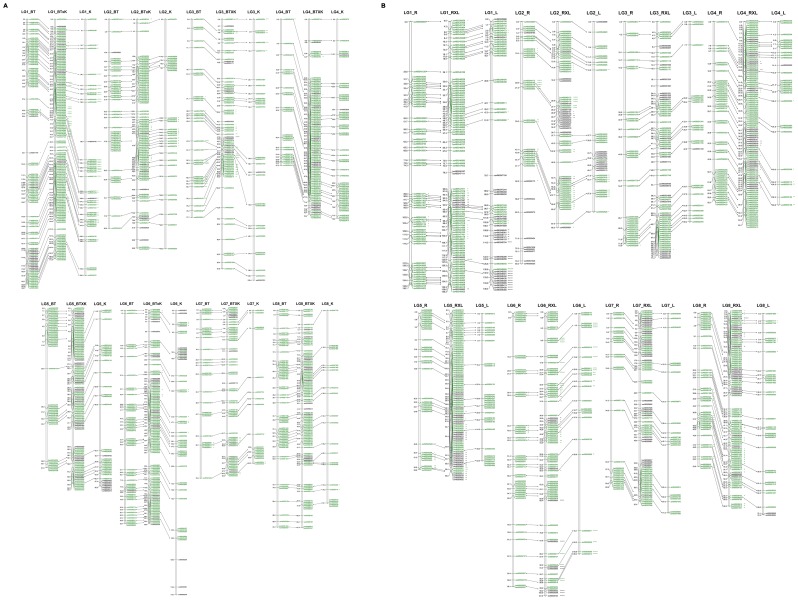
Sweet cherry highly dense linkage maps of two intraspecific progenies and their parental lines. Sweet cherry linkage maps constructed from two intraspecific progenies ‘Black Tartarian’ × ‘Kordia’ (BT×K) and ‘Regina’ × ‘Lapins’ (R×L) were created using 723 and 687 molecular markers, respectively. (a) BT×K linkage map and their parental maps (BT and K linkage maps). (b) R×L linkage map and their parental maps (R and L linkage maps). Anchored markers are indicated by connecting lines and are represented in green. Markers in black are unique to each map. Distance between markers is represented in cM. Skewed markers mapped are represented by asterisks to indicate distortion level (* for p<0.1; ** p<0.05; ***p<0.01; **** p<0.005; ***** p<0.001; ****** p<0.0005; ******* p<0.0001). See larger Figure-2-SI-Figure in the supporting information.

Based upon marker segregation in the intra-specific segregating populations, four parental linkage maps were obtained using SNPs heterozygous only in one parent to increase the precision of marker localization (Table S1). All maps contain eight LGs and cover between 600–800 cM (719.4 cM in ‘Black Tartarian’, 788 cM in ‘Kordia’, 619.4 cM in ‘Regina’ and 610.1 cM in ‘Lapins’) (Table 3). Each LG contains multiple markers ranging from 21 (LG7-’Lapins’) to 99 (LG1-’Black Tartarian’). The average distance between markers ranges from 1.2 cM (LG4-’Regina’) to 3.7 cM (LG3-’Kordia’ and LG8-’Kordia’). Maximum gap size in each LG ranged from 7.4 to 28.7 (Table 3). For all parental linkage maps, a gap greater than 25 cM was obtained in LG1 (Figure 2a, 2b; Figure S3; Table 3).

**Table 3:** Number of markers, and map size and density of each parent map and of the two consensus maps (BT×K) and (R×L).

		LG1	LG2	LG3	LG4	LG5	LG6	LG7	LG8	Total
**Number of markers**	**BT**	99	47	31	25	43	53	28	58	384
	**K**	41	30	28	51	37	47	19	22	275
	**BT×K**	**133**	**84**	**68**	**89**	**95**	**106**	**55**	**93**	**723**
	**R**	61	38	35	48	35	47	29	42	335
	**L**	56	33	22	29	30	24	21	32	247
	**R×L**	**98**	**67**	**77**	**102**	**91**	**82**	**77**	**93**	**687**
**Size (cM)**	**BT**	189.7	81.9	77.7	57.3	61.3	89.7	70.6	91.2	719.4
	**K**	184.2	89.9	102.4	77.9	68.5	119.7	64.3	81.1	788.0
	**BT×K**	**171.9**	**89.8**	**97.9**	**76.4**	**67.4**	**89.6**	**68.5**	**91.4**	**752.9**
	**R**	136.7	80.3	73.6	59.1	55.9	98.1	60.8	54.9	619.4
	**L**	136.4	63.1	66.4	60.8	52.9	86.7	71.6	72.2	610.1
	**R×L**	**136.2**	**68.3**	**77.4**	**64.1**	**56.8**	**101.5**	**66.4**	**69.2**	**639.9**
**Average marker distance (cM)**	**BT**	1.9	1.7	2.5	2.3	1.4	1.7	2.5	1.6	1.9
	**K**	4.5	3.0	3.7	1.5	1.9	2.5	3.4	3.7	3.0
	**BT×K**	**1.3**	**1.1**	**1.4**	**0.9**	**0.7**	**0.8**	**1.2**	**1.0**	**1.1**
	**R**	2.2	2.1	2.1	1.2	1.6	2.1	2.1	1.3	1.8
	**L**	2.4	1.9	3.0	2.1	1.8	3.6	3.4	2.3	2.5
	**R×L**	**1.4**	**1.0**	**1.0**	**0.6**	**0.6**	**1.2**	**0.9**	**0.7**	**0.9**
**Maximum gap size (cM)**	**BT**	26.6	11.4	9.5	10.8	15.7	13.6	13.9	10.8	14.0
	**K**	28.4	20.1	20.2	11.6	18.1	13.2	20.8	15.5	18.5
	**BT×K**	**8.5**	**11.2**	**7.7**	**9.5**	**12.0**	**7.2**	**7.4**	**9.1**	**9.1**
	**R**	25.5	11.5	14.8	7.4	12.1	14.6	18.5	7.9	14.0
	**L**	28.7	21.7	17.9	12.4	8.7	27.4	15.4	8.8	17.6
	**R×L**	**12.3**	**7.6**	**4.2**	**4.5**	**6.2**	**9.8**	**9.4**	**5.9**	**7.6**

Skewed Mendelian segregation (*p*<0.01) was highly dependent on the genotype: 3.4% (13/384) in ‘Black Tartarian’, 8.4% (23/275) in ‘Kordia’, 2.7% (9/335) in ‘Regina’ and 12.5% (31/247) in ‘Lapins’. In ‘Lapins’, all skewed markers were located on LG1 (114–136 cM) and LG6 (0–21 cM and 78–87 cM) (Figure 2b; Figure S3). For the other cultivars, skewed markers were spread in several LGs: LG1 (65–121 cM in ‘Black Tartarian’, 40–160 cM in ‘Kordia’, 96 cM in ‘Regina’), LG2 (20–21 cM) in ‘Regina’, LG3 (40–70 cM in ‘Black Tartarian’ and 0–12 cM in ‘Kordia’), LG5 (55 cM in ‘Regina’) and LG6 (5–51 cM in ‘Kordia’) (Figure 2a, 2b; Figure S3).

#### Intra-specific segregating population linkage maps

To reduce gaps in parental linkage maps, two linkage maps were constructed for each intra-specific segregating population including heterozygous markers in both parents (<hk×hk> markers). Each map consisted of eight *Prunus* LGs (Figure 2a, 2b; Figure S4). The maps covered 752.9 and 639.9 cM, containing 723 and 687 markers, respectively (Table 3). Each LG contained multiple markers, ranging from 55 to 133 markers. The average cM distance between markers was 1.1 for BT×K and 0.9 cM for R×L. LG1 was the longest group in both maps covering 171.9 cM with 133 markers in BT×K, and 136.2 cM with 98 markers in R×L. LG5 was the shortest group in both maps, covering 67.4 cM with 95 markers in BT×K, and 56.8 cM with 91 markers in R×L. Maximum gap size in each chromosome ranged from 4.2 to 12.3 (Table 3). In BT×K the largest gap (12 cM) was found in LG5 between the ss490554476 and ss490554529 markers; whereas in R×L it was in LG1 (12.3 cM), between the markers ss490547111 and ss490547271.

**Figure 3:**
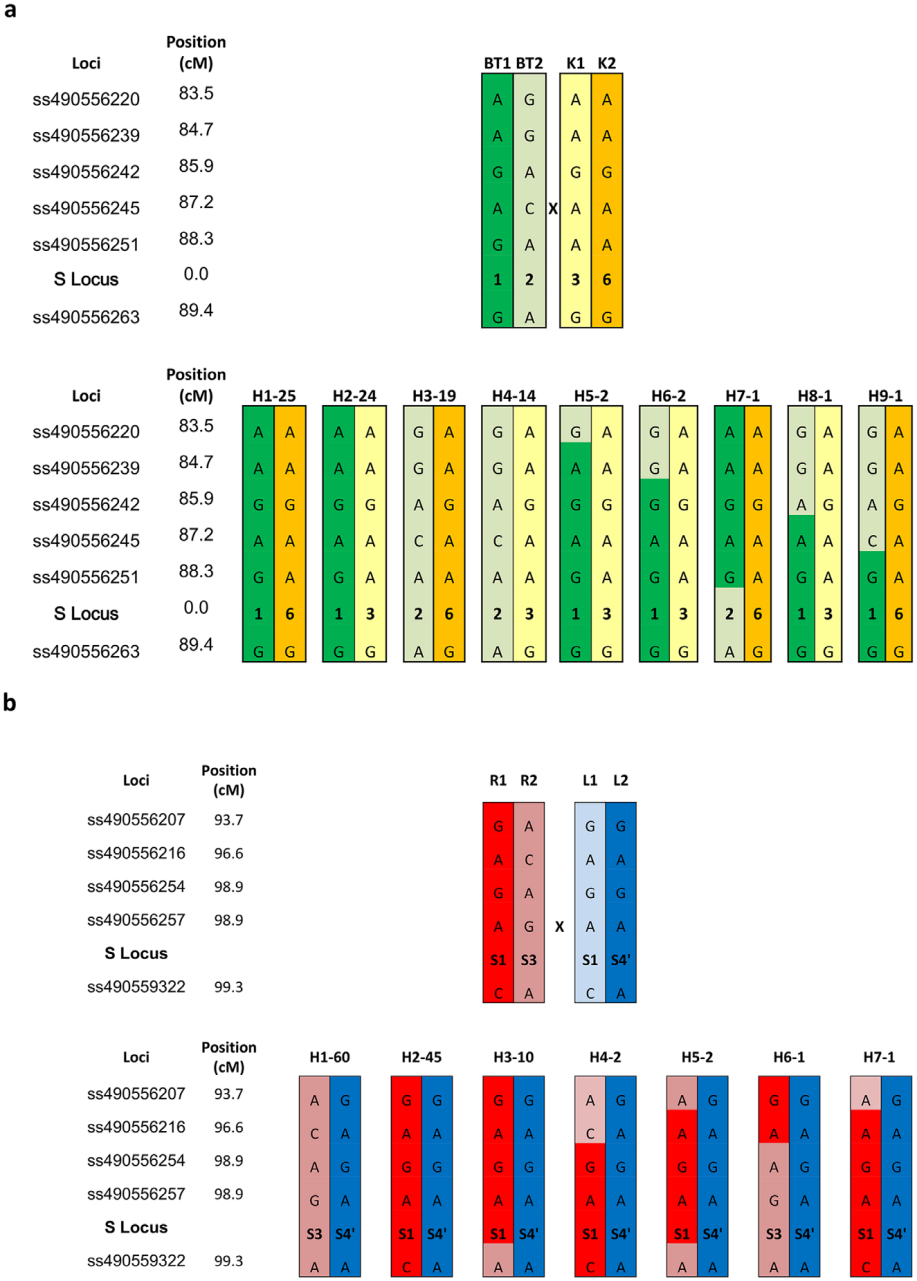
Haplotype analysis using SNPs markers spanning the self-incompatibility S locus in each population. (a) ‘Black Tartarian’ × ‘Kordia’ (BT×K) and (b) ‘Regina’ × ‘Lapins’ (R×L).

A total of 3.8% (28/723) and 3.9% (27/687) skewed markers (chi-square probability *p*<0.01) were mapped in BT×K and R×L, respectively. Similarly to the parental linkage maps, clusters of skewed markers were found on LG1 (16/28 in BT×K), LG2 (5/27 in R×L), LG3 (12/28 in BT×K) and on LG6 (20/27 in R×L) (Figure 2a, 2b; Figure S4). This last region on LG6 corresponds to the *S* locus location in *Prunus* [14]. In the R×L progeny, ‘Regina’ genotype for the S alleles being *S_1_S_3_* and ‘Lapins’ being *S_1_S_4_* (Table 1), all the individuals inherited the *S_4’_* from ‘Lapins’ as the *S_1_* is incompatible in an *S_1_* style (Figure 3). Using the markers framing the *S* locus described in Peace *et al*. ([23]), the segregation of a 6 cM segment containing the *S* locus was followed in the two mapping progenies. For three individuals, a recombination was detected between the *S_1_*, *S_3_* alleles from ‘Regina’. Within the BT×K progeny, 92.1% (82/89) individuals were characterized by non-recombinant haplotypes including the four combinations: S_1_S_6_ (25/89), S_1_S_3_ (24/89), S_2_S_6_ (19/89) and S_2_S_3_ (14/89). The other seven individuals were detected with one recombination event only in the ‘Black Tartarian’ parent (Figure 3).

#### Comparative analyses among the linkage maps

As parental maps were constructed with markers heterozygous for only one parent of the cross (i.e. either <nn × np> or <lm × ll>), markers were in common between just one parent of each cross and the two parents of the other cross. The comparison of the parental maps was based upon the position of 277 markers: 88 common makers between ‘Black Tartarian’ and ‘Regina’, 74 between ‘Black Tartarian’ and ‘Lapins’, 87 between ‘Kordia’ and ‘Regina’, and 28 between ‘Kordia’ and ‘Lapins’ (Figure S3). A perfect co-linearity of the common markers between the parental linkage maps compared was found.

Comparison between BT×K and R×L linkage maps was performed using a total 379 common markers, representing 52.4% of the markers mapped in BT×K and 55.2% of the markers mapped in R×L (Figure S4). A total of 67 RosCOS markers, present in the RosBREED cherry 6K SNP array v1, were mapped in both the BT×K and R×L linkage maps. Common markers between the BT×K and R×L maps within each LG vary from 63 to 34 markers: 63 (LG5), 60 (LG8), 54 (LG6), 51 (LG4), 44 (LG1), 37 (LG2), 36 (LG7) and 34 (LG3). The marker positions were conserved between the two sweet cherry linkage maps constructed and only small rearranged orders were found (Figure S4).

#### Comparison between the sweet cherry genetic linkage maps and the physical peach map

Anchored markers between the intra-specific sweet cherry maps (BT×K and R×L) and the peach genome revealed that the relative position of 91.8% (944/1028) SNP markers are conserved between sweet cherry and peach (Table S1), demonstrating a high level of synteny between these two species. Only a small number of markers differed in their relative position when compared to the peach physical map (Table 4).

**Table 4:** Regions in which SNP marker orientations within the LGs are different between sweet cherry parental maps and the Peach v1.0.

LG	Peach region (Mb)	Black Tartarian			Kordia			Regina				Lapins		
		ss#	Peach Physical position	cM	ss#	Peach Physical position	cM		ss#	Peach Physical position	cM	ss#	Peach Physical position	cM
1*	∼42.3– ∼46.6	ss490548680	46635504	168.6	ss490548567	45402154	179.6		ss490548597	45720052	127.5	ss490548655	46402818	130.6
		ss490548614	45924398	171	ss490559090	45226245	179.6		ss490548589	45633267	129.3	ss490548555	45210413	132.2
		ss490548575	45535084	174	ss490548551	45163766	184.2		ss490548538	45028492	132.7	ss490548501	44685238	132.2
		ss490546947	45498179	174	−	−	−		ss490548567	45402154	132.7	ss490548452	44132511	133
		ss490559325	45473668	174	−		−		ss490546919	44823707	132.7	ss490548430	43975890	133
		ss490548571	45473214	174	−	−	−		ss490548506	44719900	133.6	ss490546889	43556762	133.9
		ss490559081	45469715	174	−	−	−		ss490548497	44648246	134.4	ss490559028	43223890	133.9
		ss490546919	44823707	174	−	−	−		ss490548488	44565586	134.4	ss490559413	42901172	133.9
		ss490548510	44764542	174	−	−	−		ss490548460	44247150	134.4	ss490559428	42355743	136.4
		ss490546951	45748141	174	−	−	−		ss490546908	44564237	134.4	ss490548360	42337214	136.4
		ss490548501	44685238	174.2	−	−	−		ss490548418	43875207	135.2	−	−	−
		ss490548589	45633267	174.2	−	−	−		ss490548402	43706470	136.1	−	−	−
		ss490559189	45682217	175.5	−	−	−		−	−	−	−	−	−
		ss490548593	45680542	175.5	−	−	−		−	−	−	−	−	−
		ss490548448	44090182	180.5	−	−	−		−	−	−	−	−	−
		ss490548444	44045161	181.6	−	−	−		−	−	−	−	−	−
		ss490558979	44008562	181.6	−	−	−		−	−	−	−	−	−
		ss490558976	44008658	181.6	−	−	−		−	−	−	−	−	−
		ss490558982	44008459	181.6	−	−	−		−	−	−	−	−	−
		ss490548414	43863991	182.7	−	−	−		−	−	−	−	−	−
		ss490548418	43875207	182.7	−	−	−		−	−	−	−	−	−
		ss490558932	43832684	182.7	−	−	−		−	−	−	−	−	−
		ss490559070	43353354	185.9	−	−	−		−	−	−	−	−	−
		ss490548373	42731565	187.4	−	−	−		−	−	−	−	−	−
		ss490548360	42337214	189.7	−	−	−		−	−	−	−	−	−
4	∼26.7– ∼26.8	−	−	−	ss490553438	26771048	77.3		ss490553432	26692324	58.26	−	−	−
		−	−	−	−	−	−		ss490553438	26771048	58.26	−	−	−
5	∼0.7− ∼2.7	ss490553790	2722408	0	−	−	−		−	−	−	ss490549013	2351581	0
		ss490549013	2351581	1.13	−	−	−		−	−	−	ss490559171	1709155	3.3
		ss490559171	1709155	1.13	−	−	−		−	−	−	ss490553708	1221714	5.02
		ss490553708	1221714	2.71	−	−	−		−	−	−	ss490553696	1121958	5.86
		ss490553696	1121958	2.71	−	−	−		−	−	−	ss490553674	934368	6.7
		ss490553674	934368	2.71	−	−	−		−	−	−	ss490553668	857660	7.56
−		ss490553668	857660	3.81	−	−	−		−	−	−	ss490553641	671432	9.67
		ss490553641	671432	3.81	−	−	−		−	−	−	−	−	−
5	∼3.5– ∼4.8	−	−	−	ss490549071	4831572	0		−	−	−	−	−	−
		−	−	−	ss490553907	4413731	0		−	−	−	−	−	−
		−	−	−	ss490553832	3526608	3.14		−	−	−	−	−	−
6*	∼0.0– ∼1.3	−	−	−	−	−	−		ss490555017	1144673	0	−	−	−
6*	∼1.3– ∼4.3	−	−	−	ss490555200	4315623	5.84		−	−	−	ss490555175	4050337	0
		−	−	−	ss490555178	4054442	5.84		−	−	−	ss490555147	3425466	0
		−	−	−	ss490559372	3912624	5.84		−	−	−	ss490555075	2247834	4.17
		−	−	−	ss490555172	3973271	9.21		−	−	−	ss490549593	1852409	4.17
		−	−	−	ss490555169	3920291	9.21		−	−	−	ss490555047	1811133	4.17
		−	−	−	ss490555068	2122364	18.35		−	−	−	−	−	−
		−	−	−	ss490555062	2061776	18.35		−	−	−	−	−	
		−	−	−	ss490559096	1954309	18.35		−	−	−	−	−	−
		−	−	−	ss490555056	1955297	18.35		−	−	−	−	−	−
		−	−	−	ss490559093	1954283	18.35		−	−	−	−	−	−
		−	−	−	ss490549593	1852409	18.35		−	−	−	−	−	−
		−	−	−	ss490555047	1811133	19.48		−	−	−	−	−	−
		−	−	−	ss490549585	1734892	19.48		−	−	−	−	−	−
7*	∼0.0– ∼4.5	ss490556549	4261930	0.94	−	−	−		ss490559416	4467254	0	ss490556549	4261930	0
		ss490556476	2481336	0.94	−	−	−		ss490559378	4466543	0	ss490556476	2481336	1.65
		−	−	−	−	−	−		ss490556418	1275199	1.61	ss490556403	926005	4.9
7	∼5.5– ∼6.6	ss490556594	5729126	0	ss490556612	6594902	0		−	−	−	−	−	−
		ss490556591	5532720	0	ss490556597	5868634	0		−	−	−	−	−	−

* Region corresponding to randomly oriented scaffolds within peach V1.0 assembly (I. Verde personal communication).

The SNPs in this table have been ordered cronologically for the position (cM) of the SNP markers on the sweet cherry parental maps. The majority of SNP markers with the LGs are oriented such that the number increases, similar to the orientation seen on the peach physical map. The regions represented in this table have differences between the orientation of the SNP markers on the LG, when compared to the peach physical map. Black boxes represent regions of the LG outside of the region in question. Green boxes represent regions in which the orientation of the SNP markers are inverted in relationship to the rest of the LG as well as the peach physical map. Marker position (cM) of the parental maps compared to the physical peach position v1.0, revealed a small percentage of local discrepancies (8.2%). There was only partial co-linearity of markers within the distal regions of LG1 and LG4; as well as the proximal regions of LG5, LG6 and LG7 of sweet cherry and peach (Table 4; Figure S3; Table S1). In region ∼42.3 Mb to ∼46.6 Mb of LG1, a total of 45 markers differed in their relative position on the parental maps when compared to the peach genome (‘Black Tartarian’ = 25, ‘Kordia’ = 3, ‘Regina’ = 12, and ‘Lapins’ = 10), five of these markers demonstrate a conservation of their relative position in at least two sweet cherry parental maps. In the region between ∼26.7 Mb and ∼26.8 Mb on LG4, there were local discrepancies in two markers on the ‘Regina’ map and one local discrepancy on the ‘Kordia’ map. The relative distance of the local discrepancy detected in ‘Kordia’ is only 0.7 cM relative to the nearest marker. This difference may be due to imprecisions associated with the small population size used in creating this map (89 individuals). However, the marker was conserved between ‘Kordia’ and ‘Regina’ maps. It was not possible to confirm this discrepancy in the other two parental maps, due to a limited number of polymorphic markers in this region in the other two cultivars. Similarly, on LG5, a discrepancie were observed between ∼0.7 Mb and ∼2.7 Mb for eight and seven markers in ‘Black Tartarian’ and ‘Lapins’, respectively. Three markers also showed discrepancies in ‘Kordia’ between ∼3.5 Mb to ∼4.8 Mb. On LG6, a discrepancy between ∼0.0 Mb and ∼1.3 Mb was observed only in the ‘Regina’ map (one marker). Other discrepancy in the region between ∼1.3 Mb and ∼4.3 Mb in the same linkage group was found for 13 and five markers in ‘Kordia’ and ‘Lapins’, respectively. On LG7, in a region from ∼0.0 Mb to ∼4.5 Mb, three markers in both ‘Regina’ and ‘Lapins’ maps were placed in an “inverted order” when compared with the peach physical map. However, in ‘Black Tartarian’ only two of the three markers were found having discrepancy with the peach genome. This “inverted order” was not found in ‘Kordia’ map. Other region on LG7 has discrepancy in two of the maps (‘Black Tartarian’ and ‘Kordia’ maps), from ∼5.5 Mb to ∼6.6 Mb for two markers in each parent (Table 4; Figure S3).

Only partial co-linearity of markers within the proximal region on LG5, LG6 and LG7; and distal region on LG1 and LG4 of intra specific sweet cherry maps (BT×K and R×L) and peach were detected, similar to what we observed in the parental maps. On the other hand, 1% (10/1.028) of the SNP markers were located on different LGs in the BT×K and R×L maps than predicted based upon the peach genome (v1.0) [14] (Table 5). A comparison between the position of these ten markers in the peach genome and the sweet cherry high density linkage maps revealed a clustering of four of the SNP markers in two narrow zones on LG8 in both progenies, situated at 45.0 cM and 62.6 cM in BT×K and at 44.5 cM and ∼58.4 cM in R×L (Table 5).

**Table 5:** Sweet cherry SNP RosBREED markers located in different LGs within the sweet cherry high density linkage maps in comparison with the peach genome v1.0.

		Peach genome v1.0		BT×K		R×L	
ss#	SNP RosBREED	LG	Physical position	LG	cM	LG	cM
ss490549200	RosBREED_snp_sweet_cherry_Pp2_15181737	2	15181737	−	−	1	66.9
ss490549789	RosBREED_snp_sweet_cherry_Pp2_17791585	2	17791585	−	−	4	38.9
ss490549077	RosBREED_snp_sweet_cherry_Pp2_13301587	2	13301587	5	26.3	−	−
ss490550875	RosBREED_snp_sweet_cherry_Pp3_01008652	3	1008652	−	−	8	58.0
ss490553515	RosBREED_snp_sweet_cherry_Pp4_28593928	4	28593928	−	−	2	7.2
ss490548794	RosBREED_snp_tart_cherry_a_Pp4_16764887	4	16764887	−	−	6	62.2
ss490553195	RosBREED_snp_sweet_cherry_Pp4_19398313	4	19398313	−	−	7	61.5
ss490555352	RosBREED_snp_sweet_cherry_Pp6_06747374	6	6747374	8	62.6	8	58.4
ss490556354	RosBREED_snp_sweet_cherry_Pp6_28206268	6	28206268	8	45.0	8	44.5
ss490550342	RosBREED_snp_tart_cherry_Pp6_28201446	6	28201446	8	45.0	8	44.5

Syntenic regions between the sweet cherry high density linkage maps and the *Prunus* bin map [24] were determined by comparing the location of RosCOS markers on the maps (Figure S5). The relative positions of the markers in the BT×K and R×L intra specific sweet cherry maps (55 and 51 RosCOS markers, respectively) and the *Prunus* bin map are highly conserved, with the exception of one marker which is differential positions in both the BT×K and R×L maps when compared to the *Prunus* bin map (Figure S5, marker ss490559286 located on LG6). Interestingly, this marker is located in the region of LG6 in which there are discrepancies between the ‘Kordia’ map and the peach genome.

There is also an almost perfect conservation of the relative map positions of these RosCOS markers when comparing the BT×K and R×L intra specific sweet cherry maps with the consensus map reported by Cabrera [19]. Of a total of 39 and 45 RosCOS markers conserved between the map reported by Cabrera [19] and the BT×K and R×L intra specific sweet cherry maps, respectively, there is only one RosCOS marker (RosCOS1551) located on LG5 whose relative position is not conserved in the R×L map.

## Discussion

The number of heterozygous markers observed using the RosBREED cherry 6K SNP array v1 in each of the four parents (515–634, Table 2) represent 9–11% of all SNPs on the array (28–35% of 1,825 SNPs reported to be polymorphic among the 269 sweet cherry accessions evaluated by Peace *et al*. [23]). These results are consistent with the heterozygosity estimation of 400–700 markers for any given sweet cherry cultivar suggested by Peace *et al*. [23]. The majority of these heterozygous SNPs were SNPs that would more likely be heterozygous given that the MAFs for the majority of these SNPs were >0.2. Additionally, these results sustain the choice of the accessions used for the detection panel in the RosBREED cherry 6K SNP array v1 to efficiently represent cherry breeding germplasm from different origins (Table 1). The common gaps on chromosome 1 (∼21.0 Mb), chromosome 2 (∼8.5 Mb), chromosome 3 (∼12.0 Mb), chromosome 4 (∼24.5 Mb), chromosome 5 (∼7.0 Mb), chromosome 6 (∼15.0 Mb), chromosome 7 (∼5.0 Mb), and chromosome 8 (∼10.0 Mb), may represent putative centromeric regions (S. Scalabrin, personal communication) as it was already reported for peach [21]. These regions correspond to low recombination frequency as shown in Figure S1; S2.

A linkage map based on an intra-specific cross between ‘Regina’ and ‘Lapins’ has been previously reported [17]. Using the progeny of this intra-specific cross, as well as a newly developed intra-specific segregating population produced from a cross between ‘Black Tartarian’ × ‘Kordia’, we have constructed two high-density sweet cherry linkage maps. Over 650 SNPs have been placed on these high-density linkage maps, using the RosBREED cherry 6K SNP array v1. The vast majority of these SNP markers have not previously been positioned on sweet cherry genetic linkage maps. Additionally, analyses of the SNP markers in the intra-specific segregating population enabled us to develop linkage maps specific for the parental lines used in these crosses. These results demonstrate that the RosBREED cherry 6K SNP array v1 may be used to efficiently conduct genome-wide genotyping of sweet cherry cultivars and their progeny.

The length of four parental sweet cherry cultivars (719.4 cM ‘Black Tartarian’, 788 cM ‘Kordia’, 619.4 cM ‘Regina’ and 610.1 cM ‘Lapins’) are similar to those of other sweet cherry cultivars that have been published previously: 799.4 cM [19]; 711 cM in ‘EF’ and 565.8 cM in ‘NY’ [18]. Similarly, the length of the BT×K and R×L maps (752.9 cM and 639.9 cM respectively) were similar to that seen in the interspecific ‘Napoleon (*P. avium*) x *P. nipponica* consensus map (680 cM) [16] as well as the sweet cherry consensus linkage map developed by Cabrera et al. [19] (779.4 cM). The genetic distance determined for LG1, LG4, LG5, LG7 and LG8 are equivalent to those reported in the *Prunus* bin map (Figure S5). However, LG2, LG3 and LG6 are longer in BT×K and R×L when compared to the *Prunus* bin map.

A high co-linearity between BT×K and R×L maps was observed and only minor rearrangements of markers in small regions of the map were detected. Due to the relatively small sizes of the progeny population, minor variation in marker order between the BT×K and R×L high density linkage maps may be due to the lack of precision in the positioning of markers located less than 2 cM from each other. Alternatively, these minor variations between the maps may be due to imprecisions in the mapping of SNP markers that are heterozygous in both parents (<*hk × hk>*). The distance between the flanking markers and the heterozygous marker may alter the exact positioning of the heterozygous marker.

Among the large number of SNPs located in the high density sweet cherry linkage maps, the segregation of a small number of markers were skewed (28/723 BT×K and 27/687 R×L). Interestingly, most of these markers showed skewed inheritance in both segregating populations and mapped to similar regions of LG1 and LG6. The clustering of the skewed markers suggests that there may be zones within the LGs that contain deleterious genes. The cluster on the top of LG6 (∼4 Mb in peach v1.0 genome) in both mapping populations coincides with the position of the peach locus for male sterility [15]; [41]. In R×L, there was also a clustering of skewed markers near the bottom of LG6 (20 Mb in peach v1.0 genome) a zone that coincides with the position of the *Prunus* self-incompatibility (*S*) locus [15]. The *S* locus genotypes for ‘Regina’ and ‘Lapins’ are *S_1_S_3_* and *S_1_S_4’_*, respectively, therefore, progeny of this cross contain only *S_1_S_4’_* and *S_3_S_4’_* allele combinations. Pollen with the *S_1_* genotype cannot germinate and fertilization cannot occur [42], resulting in the absence of *S_1_S_1_* and *S_1_S_3_* allele combinations in the progeny. This explains the skewed segregation of the markers surrounding the *S* locus located near the distal end of LG6. By analyzing haplotypes in the region of the *S* locus, it was possible to predict the S-allele combination for all individuals of the two progenies (Figure 3). This is an example of application offered by the use of this cherry 6K SNP array that will be useful in breeding programs.

The high co-linearity of the maps between the two non-related intra-specific progenies increases the likelihood that the marker order conserved in these maps is indicative of the sweet cherry genome organization. Additionally, the resulting maps (four parents and two progenies) showed high co-linearity with the *Prunus* bin map (Figure S5). Previous genomic comparison studies have shown a high synteny level within the *Prunus* genus [15]. As expected, we found a high synteny between the four sweet cherry parents studied and the peach genome (v1.0), supporting the peach can be use as the model species for other *Prunus* members [13]; [43]; [44]. In addition; the reported discrepancies found on LG1, LG6 and LG7-from ∼0.0 Mb to ∼4.5 Mb- coincide with minor assembly errors which have been detected by the International Peach Genome Initiative (IPGI), consisting in randomly oriented scaffolds (Verde, personal communication). Considering the reorientation of these scaffolds, only a very limited number of markers were in a different order (within linkage group) in comparison to peach: 9/384 in ‘Black Tartarian’; 6/275 in ‘Kordia’; 2/335 in ‘Regina’ and 7/247 in ‘Lapins’, located on LG4, LG5 and part of LG7. The divergences could be attributable to mapping inaccuracy due to the low number of individuals analysed. Moreover, each of these divergences have only been found on two of four parental maps. Thus, our results confirm not only the high synteny of peach and sweet cherry, but also the high reliability of the peach genome sequence for comparative studies within the *Prunus* species [13]; [43]; [44].

In addition to the discrepancies within the linkage-group mentioned above, a few markers were not were not located on the same linkage group in both sweet cherry and peach. One marker in BT×K and six markers in the R×L maps mapped to different LGs then predicted based upon the peach genome. In addition, three markers, ss490555352, ss490556354 and ss490550342 were mapped to LG8 in both sweet cherry high density maps, whereas these markers are located on chromosome 6 of the peach genome. These four markers are located at ∼45 cM and ∼60 cM on LG8, suggesting that this region may have undergone a duplication event during the divergence between peach and sweet cherry. Sweet and sour cherry are considered the cultivated *Prunus* fruit species most distant from peach, as predicted by their divergent evolutionary origin [43]. Further studies would be required in order to verify the local discrepancies between the sweet cherry and peach genomes.

This is the first report of a linkage map using the RosBREED cherry 6K SNP array v1. As the four cultivars used in this work derive from different origins, the polymorphism observed for their progenies from genome-scanning with the RosBREED cherry 6K SNP array v1 indicates its utility for sweet cherry breeding programs with diverse germplasms. The high proportion of monomorphic SNP markers (Table 2) agrees with the monomorphic rate (58%) obtained in the 6K SNP array. The monomorphic rate of this array was higher than those found for the recently published SNP arrays on peach [21], and apple [20] probably due to the lower sequencing depth used in the 6K SNP array [23]. However, in this study we mapped ∼700 SNPs in each of the unrelated sweet cherry progenies examined.

The large number of common markers mapped in both progenies (379 markers) provides an excellent framework for comparative QTL discovery in sweet cherry. In conclusion, we have constructed two high-density sweet cherry linkage maps from two progenies using the RosBREED cherry 6K SNP array v1 which are highly co-linear with the previously published consensus sweet cherry linkage map, the *Prunus* bin map and the peach genome. The high density of heterozygous markers on these maps has the potential to not only enable high-resolution identification of QTLs in sweet cherry, but also to predict the genotypes at a specific locus linked to an agronomical interesting characters, as illustrated for the self-incompatibility S locus.

## Supporting Information

Figure S1 Distribution and physical spacing (Mbp) of SNP heterozygosity along the cherry chromosomes for the four linkage mapping parents. The distributions of heterozygous SNPs in ‘Black Tartarian’, ‘Kordia’, ‘Regina’, and ‘Lapins’ were compared to polymorphic SNP markers identified in a germplasm collection of 269 sweet cherry selections [23]. Marker locations are based on the peach physical map positions using the Peach v.1 reference genome.
(TIF)

Figure S2 Distribution and genetic spacing (cM) of SNP heterozygosity along the cherry chromosomes for the four linkage mapping parents. The distributions of heterozygous SNPs in’Black Tartarian’, ‘Kordia,’ ‘Regina’, and ‘Lapins’ were compared to polymorphic SNP markers identified in a germplasm collection of 269 sweet cherry selections. Marker locations are based on estimated genetic distances according to the cherry linkage map of Cabrera et al. [19] to provide common genetic locations for the polymorphic SNPs across all four parental linkage maps and genetic positions for those markers that are homozygous in at least one mapping parents.
(TIF)

Figure S3 Comparison between the four parental linkage maps (‘Black Tartarian’, ‘Kordia’, ‘Regina’ and ‘Lapins’). Distance between markers is represented in cM. Black boxes represent inconsistencies with peach genome v1.0 physical order. Markers grouped in a different LG in comparison with peach genome v1.0 are inderlined.
(TIF)

Figure S4 Comparison of the two consensus sweet cherry highly dense linkage maps of two intraspecific progenies (BT×K and R×L). Anchored markers are indicated by connecting lines and are represented in green. Markers in black are unique to each map. Distance between markers is represented in cM. Markers grouped in a different LG in comparison with peach genome v1.0 are underlined. Skewed markers mapped are represented by asterisks to indicate distortion level (* for p<0.1; ** p<0.05; *** p<0.01; **** p<0.005; ***** p<0.001; ****** p<0.0005; ******* p<0.0001).
(TIF)

Figure S5 Comparison between the sweet cherry highly dense linkage maps (BT×K and R×L) and the *Prunus* bin map. Positions of anchor loci between maps are indicated by connecting lines and are represented in green. Distance between markers is represented in cM. Markers grouped in a different LG in comparison with peach genome v1.0 are underlined. Skewed markers mapped are represented by asterisks to indicate distortion level (* for p<0.1; ** p<0.05; *** p<0.01; **** p<0.005; ***** p<0.001; ****** p<0.0005; ******* p<0.0001). Bin map markers are situated at the bottom of their corresponding bin.
(TIF)

Table S1 SNPs used in this manuscript.
(XLSX)
