# Correction: Construction and Comparative Analyses of Highly Dense Linkage Maps of Two Sweet Cherry Intra-Specific Progenies of Commercial Cultivars

**DOI:** 10.1371/annotation/80cd091d-31b2-473a-b028-ea9b211b5d9d

**Published:** 2014-01-03

**Authors:** Carolina Klagges, José Antonio Campoy, José Quero-García, Alejandra Guzmán, Levi Mansur, Eduardo Gratacós, Herman Silva, Umesh R. Rosyara, Amy Iezzoni, Lee A. Meisel, Elisabeth Dirlewanger

The most recent version of Table 4 was not included in the final manuscript. Please see the correct Table 4 here: 

**Figure:**
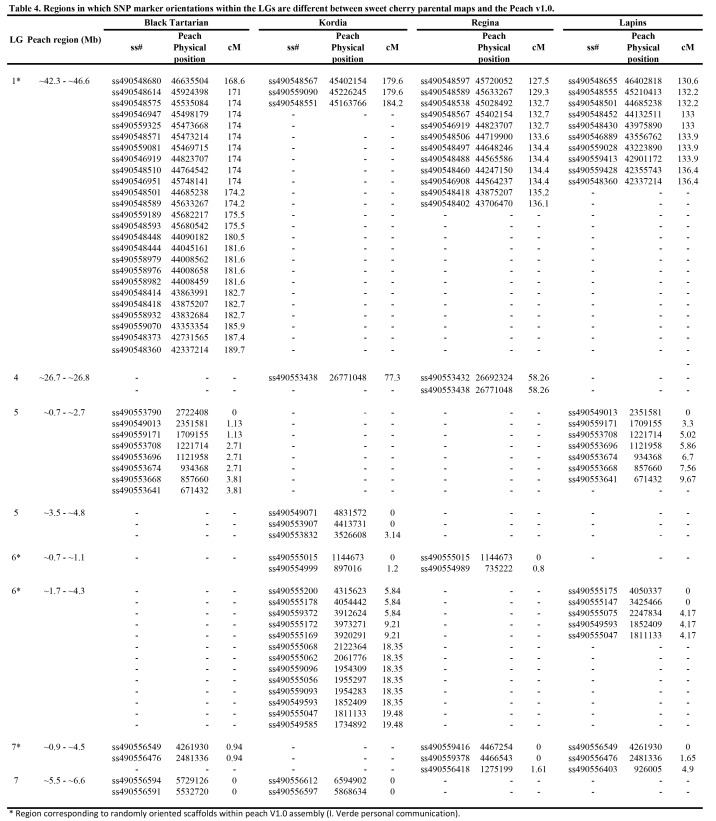


The current Table S1 mistakenly contains all the markers of the RosBREED SNP array. Please find the corrected Table S1 which only includes markers used in the manuscript here: 

The wrong version of Figure S2 was included in the final article. Please see the correct Figure S2 here: 
