# Supplementary figures and images for: Construction and Comparative Analyses of Highly Dense Linkage Maps of Two Sweet Cherry Intra-Specific Progenies of Commercial Cultivars
Source: PLoS One. 2013 Jan 31;8(1):e54743. doi: 10.1371/journal.pone.0054743 (PMC3561380; doi:10.1371/journal.pone.0054743)

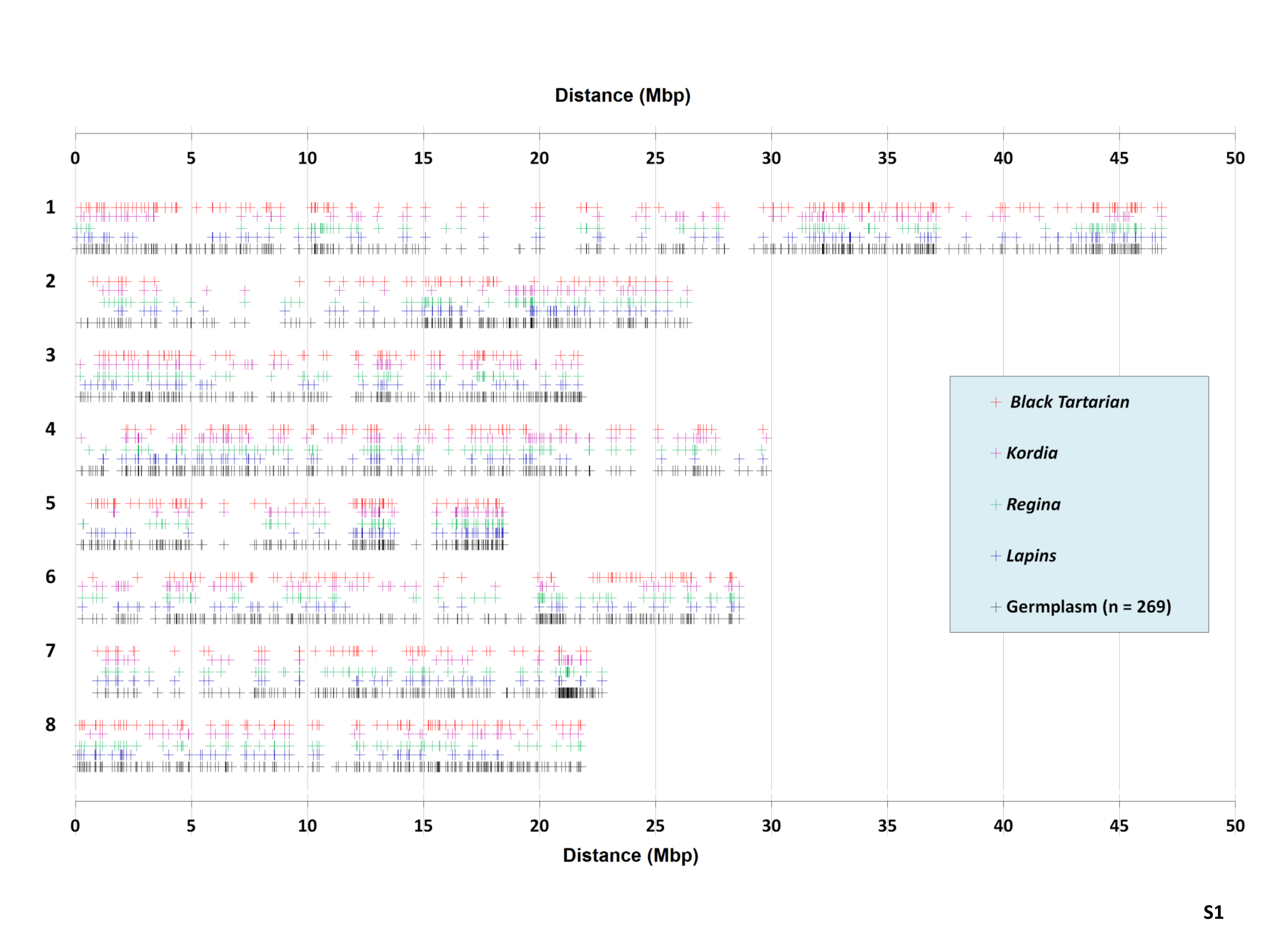

Supplement: Figure S1 — Distribution and physical spacing (Mbp) of SNP heterozygosity along the cherry chromosomes for the four linkage mapping parents. The distributions of heterozygous SNPs in ‘Black Tartarian’, ‘Kordia’, ‘Regina’, and ‘Lapins’ were compared to polymorphic SNP markers identified in a germplasm collection of 269 sweet cherry selections [23]. Marker locations are based on the peach physical map positions using the Peach v.1 reference genome. (TIF) [file pone.0054743.s001.tif]

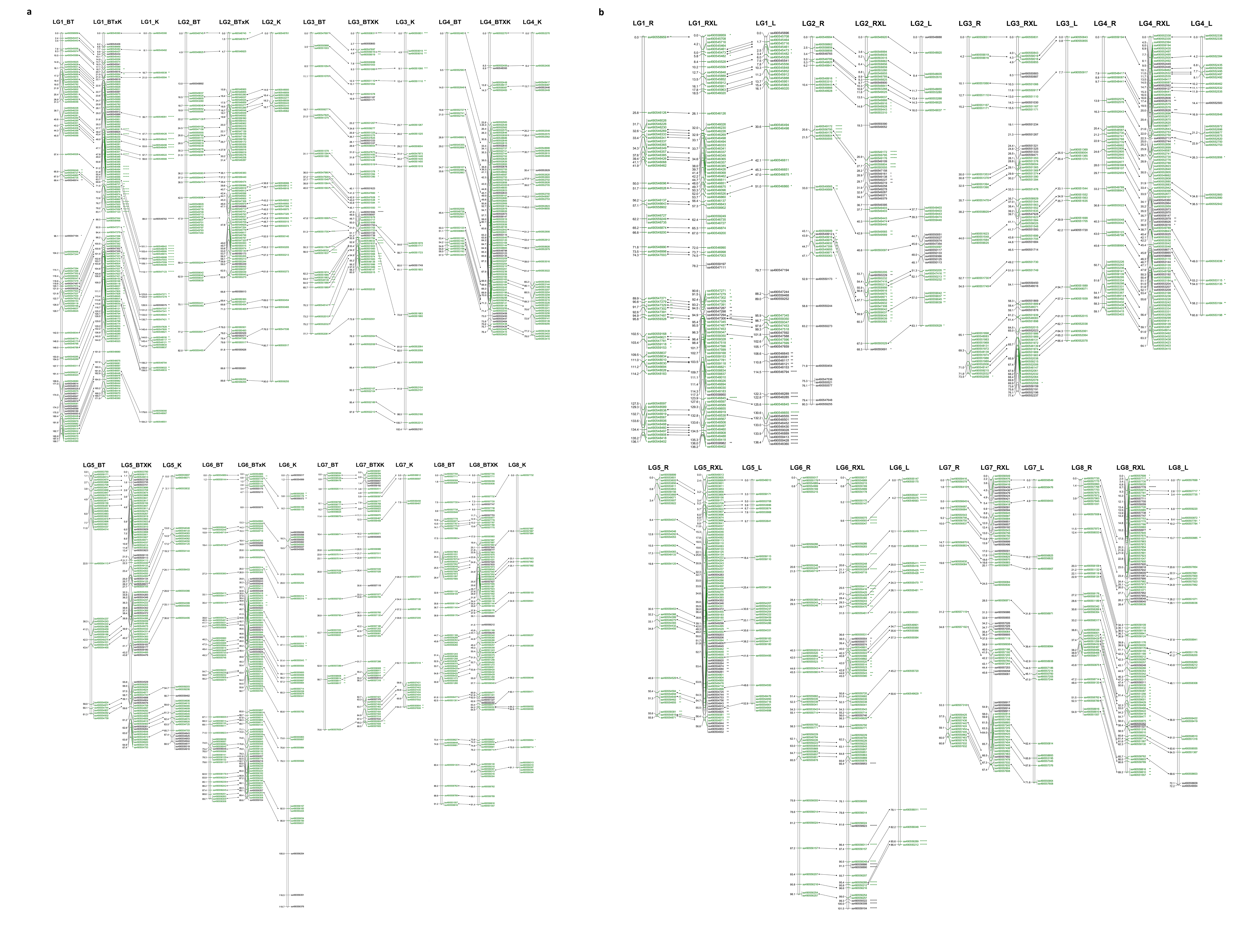

Supplement: Figure S2 — Distribution and genetic spacing (cM) of SNP heterozygosity along the cherry chromosomes for the four linkage mapping parents. The distributions of heterozygous SNPs in’Black Tartarian’, ‘Kordia,’ ‘Regina’, and ‘Lapins’ were compared to polymorphic SNP markers identified in a germplasm collection of 269 sweet cherry selections. Marker locations are based on estimated genetic distances according to the cherry linkage map of Cabrera et al. [19] to provide common genetic locations for the polymorphic SNPs across all four parental linkage maps and genetic positions for those markers that are homozygous in at least one mapping parents. (TIF) [file pone.0054743.s002.tif]

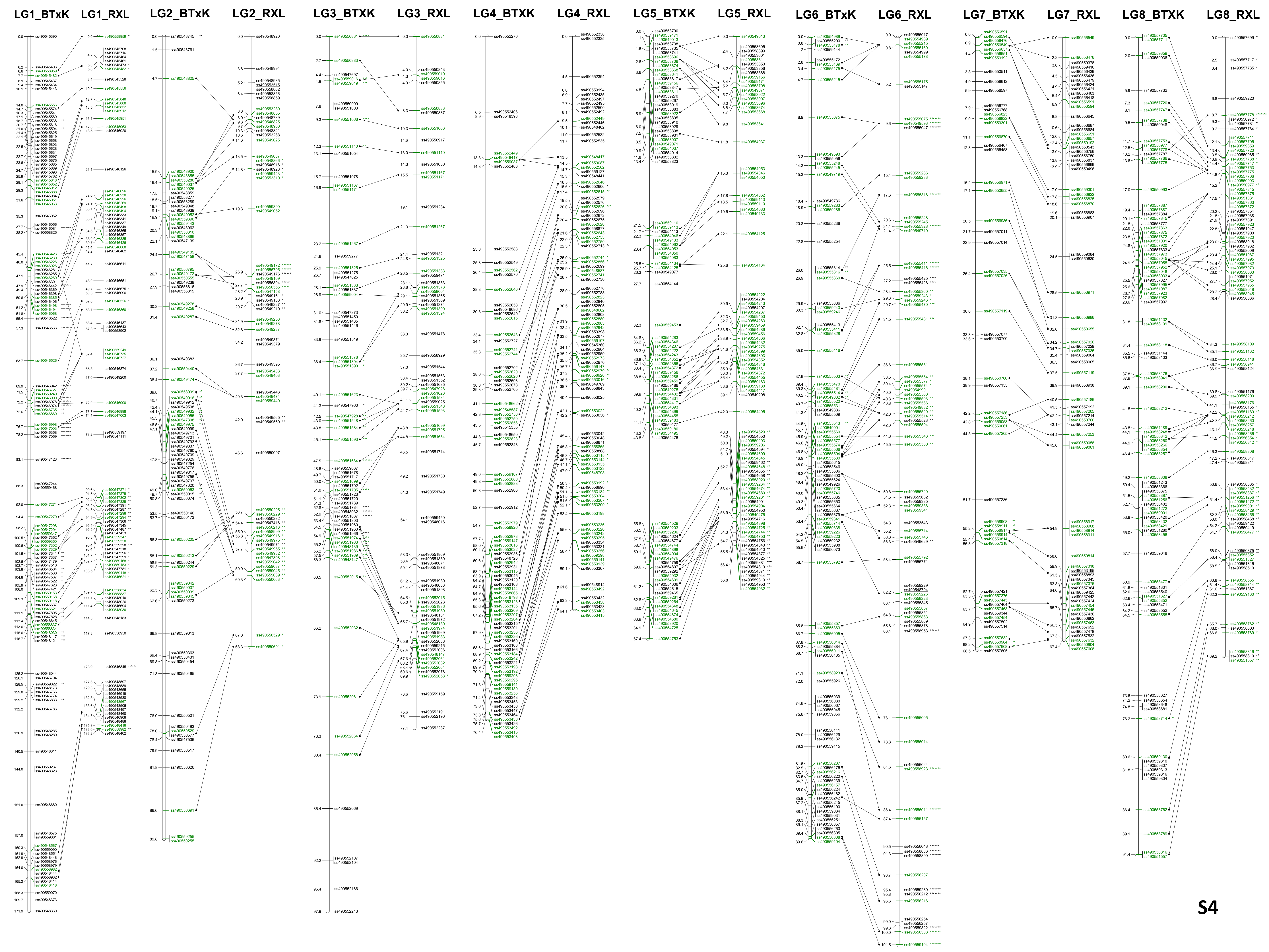

Supplement: Figure S4 — Comparison of the two consensus sweet cherry highly dense linkage maps of two intraspecific progenies (BT×K and R×L). Anchored markers are indicated by connecting lines and are represented in green. Markers in black are unique to each map. Distance between markers is represented in cM. Markers grouped in a different LG in comparison with peach genome v1.0 are underlined. Skewed markers mapped are represented by asterisks to indicate distortion level (* for p<0.1; ** p<0.05; *** p<0.01; **** p<0.005; ***** p<0.001; ****** p<0.0005; ******* p<0.0001). (TIF) [file pone.0054743.s004.tif]
