# Supplementary figures and images for: Correction: Construction and Comparative Analyses of Highly Dense Linkage Maps of Two Sweet Cherry Intra-Specific Progenies of Commercial Cultivars
Source: PLoS One. 2014 Jan 3;9(1):10.1371/annotation/80cd091d-31b2-473a-b028-ea9b211b5d9d. doi: 10.1371/annotation/80cd091d-31b2-473a-b028-ea9b211b5d9d (PMC3888323; doi:10.1371/annotation/80cd091d-31b2-473a-b028-ea9b211b5d9d)

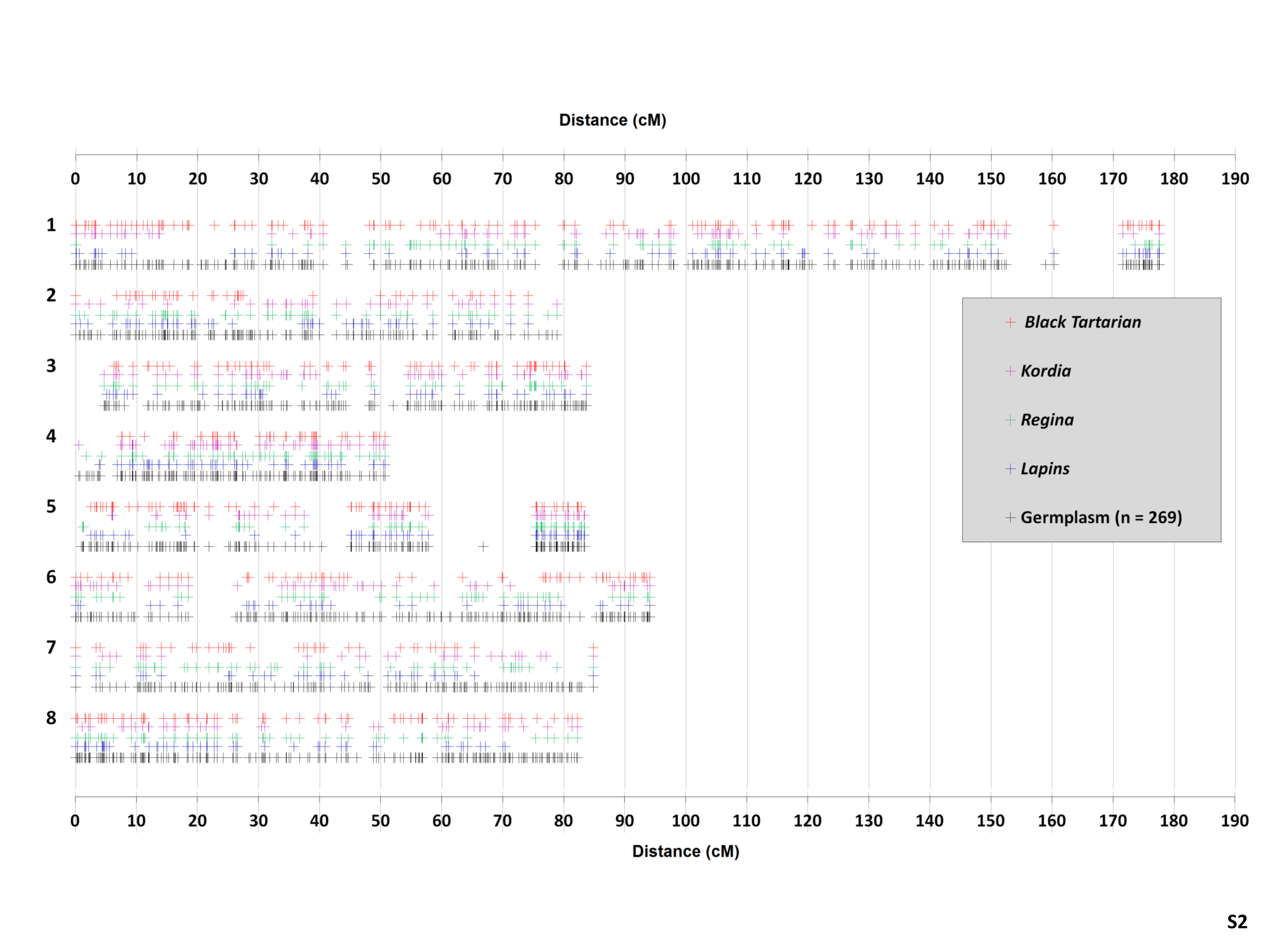

Supplement: Supplementary file 2 [file pone.80cd091d-31b2-473a-b028-ea9b211b5d9d.s002.tif]
